# Remodeling of Tumor Microenvironment by Nanozyme Combined cGAS–STING Signaling Pathway Agonist for Enhancing Cancer Immunotherapy

**DOI:** 10.3390/ijms241813935

**Published:** 2023-09-11

**Authors:** Wenpei Dong, Mengting Chen, Chun Chang, Tao Jiang, Li Su, Changpo Chen, Guisheng Zhang

**Affiliations:** 1 Electronic S Collaborative Innovation Center of Henan Province for Green Manufacturing of Fine Chemicals, Key Laboratory of Green Chemical Media and Reactions, Ministry of Education, Henan Normal University, Xinxiang 453007, China; dongwenpei@htu.edu.cn (W.D.); jiangtao@htu.edu.cn (T.J.); suli@htu.edu.cn (L.S.); 2 Henan Key Laboratory of Green Chemical Media and Reactions, School of Chemistry and Chemical Engineering, Henan Normal University, Xinxiang 453007, China

**Keywords:** cancer immunotherapy, cGAS/STING, Co,N-doped carbon dots, nanozymes

## Abstract

Nanozymes and cyclic GMP-AMP synthase (cGAS) the stimulator of interferon genes (STING) signaling pathway, as powerful organons, can remodel the tumor microenvironment (TME) to increase efficacy and overcome drug resistance in cancer immunotherapy. Nanozymes have the potential to manipulate the TME by producing reactive oxygen species (ROS), which lead to positive oxidative stress in tumor cells. Cyclic dinucleotide (2′,3′-cGAMP), as a second messenger, exists in the TME and can regulate it to achieve antitumor activity. In this work, Co,N-doped carbon dots (CoNCDs) were used as a model nanozyme to evaluate the properties of the anti-tumor mechanism, and effective inhibition of S180 tumor was achieved. Based on CoNCDs’ good biocompatibility and therapeutic effect on the tumor, we then introduced the cGAS–STING agonist, and the combination of the CoNCDs and STING agonist significantly inhibited tumor growth, and no significant systemic toxicity was observed. The combined system achieved the enhanced tumor synergistic immunotherapy through TME reprogramming via the peroxidase-like activity of the CoNCDs and cGAS–STING signaling pathway agonist synergistically. Our work provides not only a new effective way to reprogram TME in vivo, but also a promising synergic antitumor therapy strategy.

## 1. Introduction

Currently, one of the most common causes of death around the world is cancer [1]. Conventional cancer treatments have some drawbacks, including poor therapeutic efficacy, excessive dose administration, intrusive intervention, and substantially adverse effects [2,3,4,5,6]. Based on the powerful augmentation of innate and adaptive immunity, the use of the immune system to accurately identify and eradicate cancer cells, known as cancer immunotherapy, has become a rising star in the field of cancer therapy [7,8,9,10,11,12,13,14,15]. Despite tumor immunotherapy’s bright future, several difficulties still exist. The immunosuppressive tumor microenvironment (TME), which reduces the effectiveness of immune responses, is a significant barrier [16,17,18]. Therefore, outlining a global TME remodeling strategy is essential for improving cancer immunotherapy.

The TME is characterized by a highly dynamic, ever-evolving system, but it is rather challenging to accurately predict [19,20]. The TME functions as a nutrient-rich soil that provides nourishment for tumor cell proliferation, following tumor reconstruction while limiting anti-immunity. Nanozymes are nanomaterials that exhibit enzyme-like activities, which have emerged as promising candidates for TME modulation for improved cancer immunotherapy [21,22,23]. The modulation mechanism using nanozymes is based on their interference with molecular components of the TME, such as reactive oxygen species (ROS) [24]. ROS are crucial for maintaining redox reactions’ homeostasis and the modulatory influence on cell survival and signaling. Nanozymes that produce ROS, including peroxidase-like activity (POD) and oxidase-like activity (OXD), can increase the amount of reactive oxygen molecules, which can kill tumor cells [25,26]. ROS-generating nanozymes can be used to convert the immune-suppressive TME into the immune-active TME. To increase the effectiveness of the immune response, novel immuno-activating strategies should be combined with nanozymes.

Inspired by DNA damage caused by ROS, small fragments from the DNA damage could activate the cyclic GMP-AMP synthase-stimulator of interferon genes (cGAS–STING), and this pathway activation could result in TME remodeling to achieve an immunotherapy effect [27]. Type I IFNs and other inflammatory cytokines are activated to induce innate immunity, and the cGAS–STING pathway is a crucial cytosolic DNA sensing mechanism [28,29,30]. In this scenario, cGAS recognizes the abnormal DNA and swiftly reacts to activate STING, which in turn triggers a cascade reaction that kills cancer cells through an innate immune response. STING is an adapter in this scenario and inherits the activation signal from cGAS [31]. Together, the combined cGAS–STING pathway and nanozymes that induct TME reprogramming may open the door for the creation of novel therapeutic approaches.

**Figure 1:**
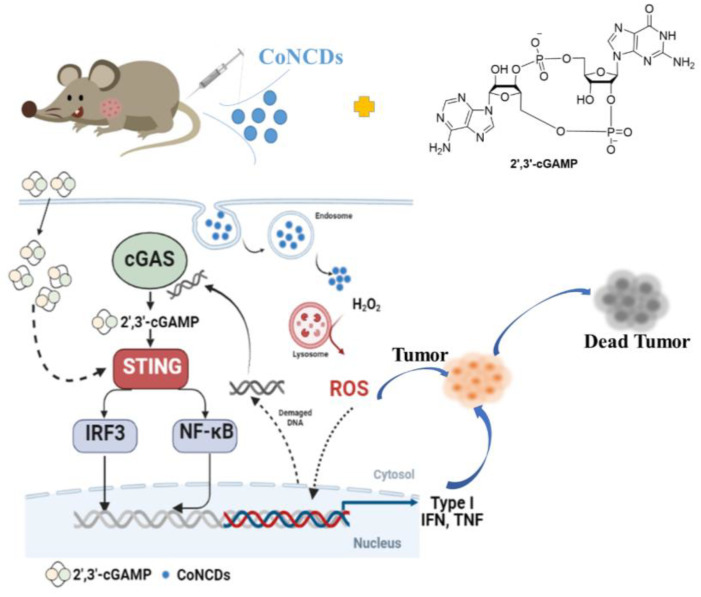
The scheme of antitumor immune responses induced by CoNCDs combined with 2′,3′-cGAMP.

Therefore, this work exploited a synergistic strategy of a nanozyme combined cGAS–STING pathway agonist to block the immunosuppression of TME. (Figure 1). Herein, we synthesized Co,N-doped carbon dots with peroxidase-like activity, which could catalyze hydrogen peroxide, trigger ROS production in the TME, and cause intracellular DNA damage to kill cancer cells. The damaged DNA might activate cGAS to generate 2′,3′-cGAMP, which could in turn activate STING. Simultaneously, the addition of 2′,3′-cGAMP can amplify the autoimmune function and increase the secretion of related cytokines, thus enhancing the anti-tumor effect. We present the first report on the combinatorial nanozymes and activation of the cGAS–STING pathway, which will be employed for the immunomodulation of the TME. This research is anticipated to offer a novel, viable solution for reprogramming the TME to improve cancer immunotherapy.

## 2. Results

### 2.1. Characterization of CoNCDs

**Figure 2:**
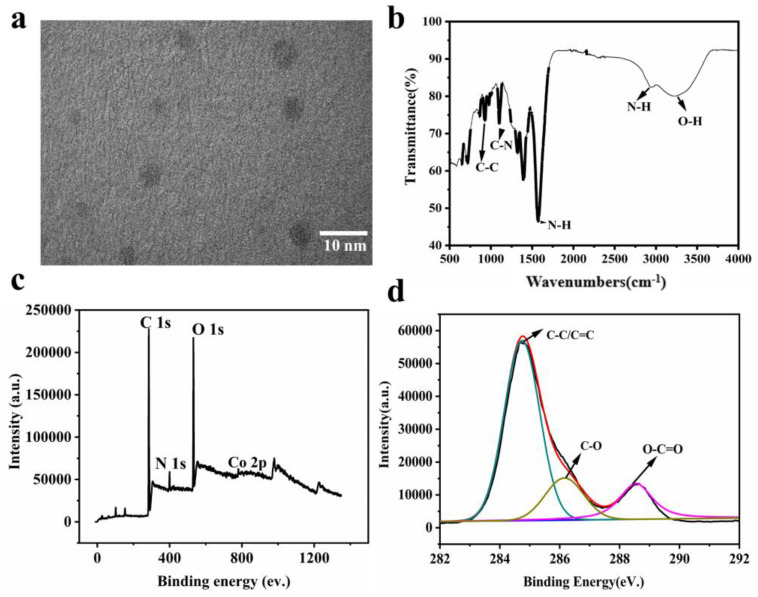
(**a**) TEM image of CoNCDs; (**b**) the FT-IR spectra of CoNCDs; (**c**,**d**) the XPS pattern of CoNCDs.

The CoNCDs were synthesized according to our previous reports [32]. To fabricate the Na_2_[Co (EDTA)] precursor, Co (NO_3_)_2_ and EDTA-Na were combined in the first step. Second, Co,N-doped carbon dots (CoNCDs) were produced through pyrolysis at 300 °C in a N_2_ environment. The synthesis yield was 8.69 ± 0.81%. Images from transmission electron microscopy (TEM) revealed that CoNCDs had a consistent, well-distributed shape and a size of 5.55 ± 1.16 nm (Figure 2a). As can be seen from the photos (Figure S1), CoNCDs could be evenly dispersed in water; the solution was a clarified system without agglomeration and precipitation from 0.5 mg/mL to 5 mg/mL. The broad peak at 3200 cm^−1^ in the FT-IR spectrum corresponded with the O-H stretching vibration, and a peak at 992 cm^−1^ revealed C-C stretching vibration, which was consistent with the results of XPS. A weak absorption peak at 2980 cm^−1^ and the medium absorption peaks at 1580 cm^−1^ were the stretching vibration and bending vibration of N-H, respectively. The peak at 1120 cm^−1^ corresponded with the C-N stretching vibration (Figure 2b), resulting in amino groups being present on the surface of CoNCDs. We also found that CoNCDs exhibited the positive charge of 1.56 ± 0.54 mV (Figure S2). Additionally, the full scan XPS spectrum of CoNCDs displayed the four characteristic peaks at 783.1 eV, 528.8 eV, 282.5 eV, and 399.4 eV, corresponding to Co (2p), O(1s), C(1s), and N(1s), respectively (Figure 2c), and the following XPS atomic percentages were achieved: Co2p (0.72%), O1s (23.28%), C1s (71.78%), and N1s (4.22%). In the C 1s spectrum (Figure 2d), there are three main peaks at 288.5 eV, 286.5 eV, and 285.0 eV, which correspond to O-C=O, C-O, and C=C/C-C. The X-ray diffraction (XRD) pattern revealed an amorphous structure for the CoNCDs (Figure S3). The Q band corresponding to the Co-coordination component was identified in the UV-Vis absorption spectrum of CoNCDs as the source of a large absorption band in the 500–600 nm region (Figure S4). At 430 nm, CoNCDs’ fluorescence emission spectra were detected at an excitation wavelength of 360 nm (Figure S5).

### 2.2. POD-like Activity of CoNCDs

**Figure 3:**
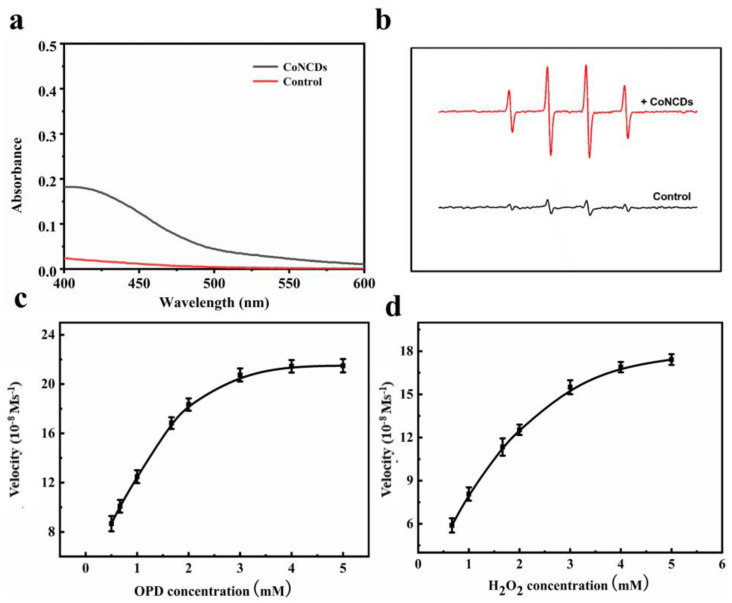
The POD-like activity of CoNCDs: (**a**) the UV-vis spectra of OPD and H_2_O_2_ in the presence of CoNCDs; (**b**) electron spin resonance (ESR) spectra of CoNCDs in the presence of H_2_O_2_; (**c**) the velocity (v) of the reaction was measured with the change of OPD concentration (3.3 mM H_2_O_2_) at pH 7; (**d**) the velocity (v) of the reaction was measured with the change of H_2_O_2_ concentration (3.3 mM OPD). The error bars represent the standard deviation of three measurements.

By accelerating the oxidation of o-phenylenediamine (OPD) in the presence of H_2_O_2_, CoNCDs displayed peroxidase-like activity (Figure 3a). In contrast to the system of OPD + H_2_O_2_, the CoNCDs + OPD + H_2_O_2_ system displayed significant absorbance, which suggests CoNCDs have peroxidase-like activity. Due to their POD-like activity, CoNCDs could catalyze the oxidation of H_2_O_2_ to produce free radicals. To determine the type of ROS produced by CoNCDs, DMPO was used to capture •OH. The four ROS signals from H_2_O_2_ that were visible in the ESR spectra showed that the kind of ROS produced was presumably •OH (Figure 3b). It was surmised that the CoNCDs could catalyze H_2_O_2_ in the TME to produce ROS. Moreover, the Michaelis–Menten constant (*K_m_*) was discovered to assess the catalytic performance of CoNCDs in the degradation of H_2_O_2_. For H_2_O_2_, the *K_m_* value was 1.26 mM, while for OPD, it was 1.06 mM (Figure 3c,d and Table S1). The peroxidase activity of CoNCDs was further demonstrated.

### 2.3. The Cellular Uptake Efficiency of CoNCDs

**Figure 4:**
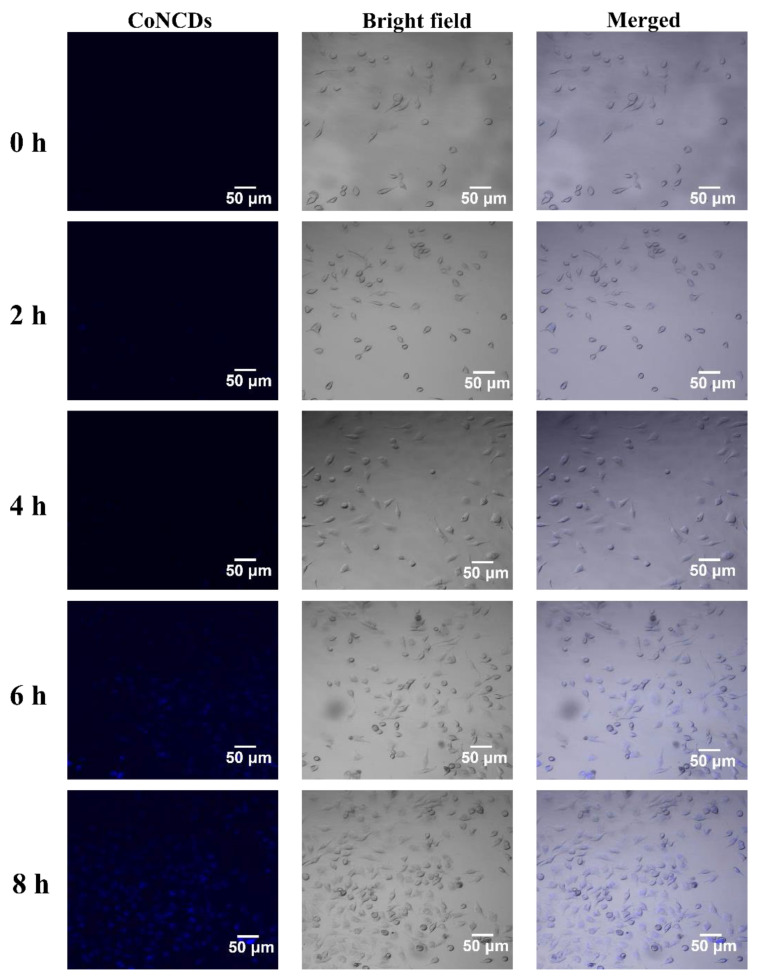
Fluorescence images of Hela cells incubated with CoNCDs at 1 h, 2 h, 4 h, 6 h, and 8 h.

Confocal microscopy was utilized to trace the blue fluorescence of CoNCDs to confirm the effectiveness of Hela cells’ uptake. CoNCDs possessed considerable transmembrane ability, as seen in Figure 4. At 2 h, blue fluorescence began to appear in the cells, indicating that CoNCDs began to enter the cells. As time went on, more and more CoNCDs entered the cells, blue fluorescence covered all the cells, and endocytic efficiency peaked at 8 h.

### 2.4. The Effect of Killing Cancer Cells with CoNCDs

**Figure 5:**
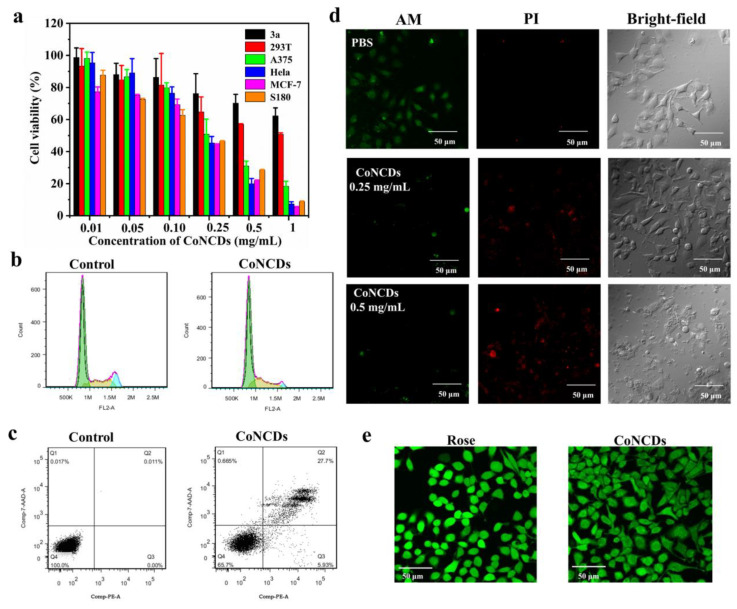
(**a**) Viability assays of different tumor cells with CoNCDs (3a, 293T, A375, Hela, MCF-7, S180 cells); (**b**) apoptosis analysis of CoNCDs; (**c**) cell cycle analysis of CoNCDs; (**d**) live/dead staining images subjected to different treatments; (**e**) intracellular ROS staining images of CoNCDs.

To ensure the effect of killing cancer cells, we measured the toxicity of CoNCDs for cancer cells and normal cells using MTT assays. According to Figure 5a, CoNCDs showed cytotoxic effects on cancer cells (MCF-7, HeLa, A375, and S180 cells.). When CoNCD concentration was increased, the percentage of cell survival declined; at a dose of 1 mg/mL, the rate of cell survival was only 20%. The IC_50_ value of 185 ± 62 μg/mL with S180 cells also confirmed the excellent anticancer efficacy of CoNCDs (Table S2). The anticancer effect of nanozymes was similar to existent nanozymes tested in cancer therapy. Comparatively, CoNCDs showed extremely low cytotoxicity against normal cells (293T and 3a normal cells), and even at high concentrations (up to 1 mg/mL), cell viability was greater than 50%. As a result, CoNCDs were found to be more hazardous to cancer cells than to healthy cells.

To investigate the underlying basis of the reduction in viability of CoNCDs, the cell cycle distribution and the apoptosis of CoNCDs were determined. According to the cell cycle analysis (Figure 5b), the percentage of cells in the G0/G1 phase observed in CoNCDs (55.86%) is much lower than that in control cells (70.00%). This is accompanied by a significant increase in the number of cells in the S phase (28.23% in CoNCDs and 19.97% in control) and in the G2 phase (15.91% in CoNCDs and 10.02% in control).

Additionally, according to the results of the apoptosis analysis displayed in Figure 5c, CoNCDs can induce apoptosis in Hela cells because their percentage of apoptotic cells (27.7%) is much higher than that of control cells (0.01%). Together, CoNCDs could cause apoptosis and arrest cell cycle in the G2/M and S phases.

To prove the effect of killing cancer cells with CoNCDs, AM/PI staining was used for visual observation. As depicted in Figure 5d, in comparison to the control group, the cell morphology of the groups treated with CoNCDs began to change. With the increased concentration of CoNCDs, a large number of disrupted or lysed cells appeared. At the same time, we discovered that there were no dead cells in the control group. The red fluorescence increased and the green fluorescence started to decrease as CoNCDs were added. The high-dose group experienced a greater reduction in cancer cells than the low-dose group did, thus exhibiting effective cytotoxic activity.

### 2.5. Intracellular ROS Detection In Vitro

ROS generated with nanozymes in the TME was the main cause of cell death, so we explored the ROS level after CoNCDs passed into cells. To verify this hypothesis, we used a probe (DFCH-DA) to detect the ROS production in Hela cells. As shown in Figure 5e, compared to the positive group of Rousp, the bright green fluorescence was detected after Hela cells were treated with CoNCDs. This indicated that CoNCDs could produce ROS in cancer cells, and the ROS was the source of anti-tumor effects.

### 2.6. In Vivo Biocompatibility of CoNCDs

The biocompatibility of CoNCDs was examined before they were applied in vivo. Firstly, mouse erythrocytes were used to assess the hemolytic toxicity of CoNCDs. The hemolytic rates of CoNCDs are all less than 30% in 1 mg/mL or less, as shown in Figure S6, demonstrating the low hemolytic toxicity of CoNCDs (Figure S6a).

ICP-MS was used to evaluate the amount of cobalt (Co) in the various organs, including the lung, heart, spleen, kidney, and liver since CoNCDs contain the heavy metal cobalt ion. The heart, liver, lung, and kidney are the main locations of CoNCDs, and the amount of Co in the liver was reduced after 24 h (Figure S6b,c). Furthermore, hematoxylin and eosin (H&E) staining was used to examine the organs. In tissue slices, no evident inflammatory lesions or tissue damage were found (Figure S6d).

### 2.7. In Vivo Antitumor Effect of CoNCDs

**Figure 6:**
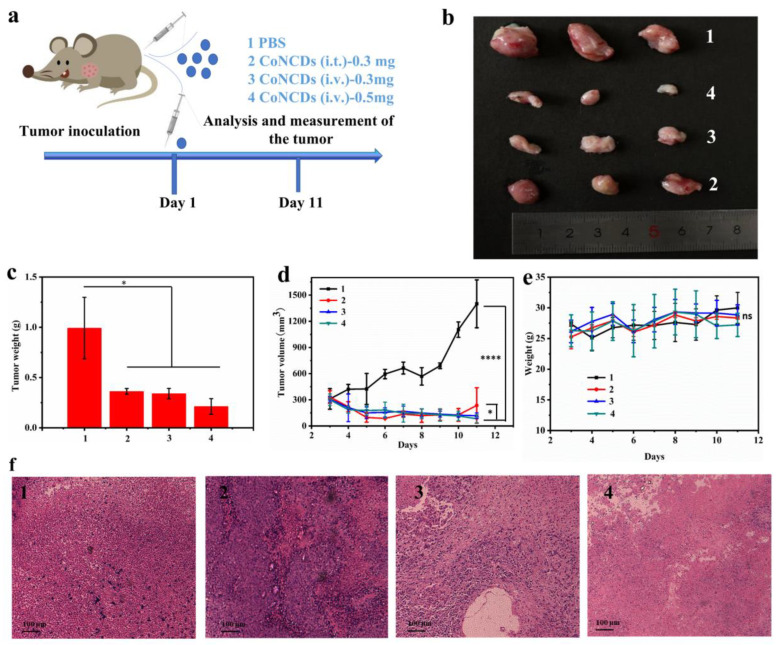
Injected CoNCDs for S180 tumor therapy: (**a**) schematic illustration of the experimental; (**b**) picture of tumors in different treatments; (**c**) tumor weight of different treatments; (**d**) tumor growth curves of different treatments; (**e**) average body weights; (**f**) tumor tissues of different groups on the 11th day were observed under a standard optical microscope (10 × 10) after H&E staining. **1**: PBS; **2**: CoNCDs were intratumorally injected (CoNCDs = 0.3 mg); **3**: CoNCDs were intravenously injected (CoNCDs = 0.3 mg); and **4**: CoNCDs were intravenously injected (CoNCDs = 0.5 mg). * *p* < 0.05, **** *p* < 0.0001, ns means no significant difference.

Inspired by the good biocompatibility and in vitro therapeutic effect of CoNCDs, we further investigated the therapeutic effect of CoNCDs in vivo with S180 tumor-bearing mice (Figure 6a). The mice were given the corresponding treatment in four groups, once the volume reached 100~200 mm^3^: (group **1**) PBS; (group **2**) CoNCDs were intratumorally injected (CoNCDs = 0.3 mg); (group **3**) CoNCDs were intravenously injected (CoNCDs = 0.3 mg); and (group **4**) CoNCDs were intravenously injected (CoNCDs = 0.5 mg). On the 11th day, the tumor volume of the PBS group was above 1000 mm^3^ and all mice were sacrificed following ethical requirements. In comparison to the control group, all the treated groups of CoNCDs (groups **2**–**5**) showed an anti-tumor effect. Following the injection of CoNCDs, tumor growth showed a decrease over time in all the CoNCD-injected groups, all of which showed strong tumor inhibition (Figure 6b–d and Figure S7). The volume of tumor in group **1** (1399.7 mm^3^) was 16 times that in group **4** (82.4 mm^3^), and the difference in tumor size could be clearly seen from Figure 6b. There was no significant difference in tumor size between group **2** and group **3**, and the tumor weight of the two groups was similar.

As observed from the tumor growth curve, the tumor volume showed a downward trend after the administration of CoNCDs (Figure 6d). Group **1** increased continuously and at a relatively high growth rate. The volumes of groups **2**–**4** were all below 250 mm^3^ on day 11. CoNCDs inhibited tumor growth and were most effective when given in high doses. Otherwise, the body weight of mice fluctuated less (Figure 6e). Subsequently, histological analysis of the tumor tissues of the mice in each group showed more necrosis and apoptosis of the tumor tissues in groups **2**–**4** compared to group **1** (Figure 6f). In addition, the tissue state was the sparsest, the nuclear dissolution was the highest, and the tissue morphology was not complete in group **4**. This also confirmed that CoNCDs had a killing effect on tumor cells, and the killing effect was the best in a large dose.

### 2.8. In Vivo Antitumor Immunotherapy Effect of CoNCDs Combined with 2′,3′-cGAMP

**Figure 7:**
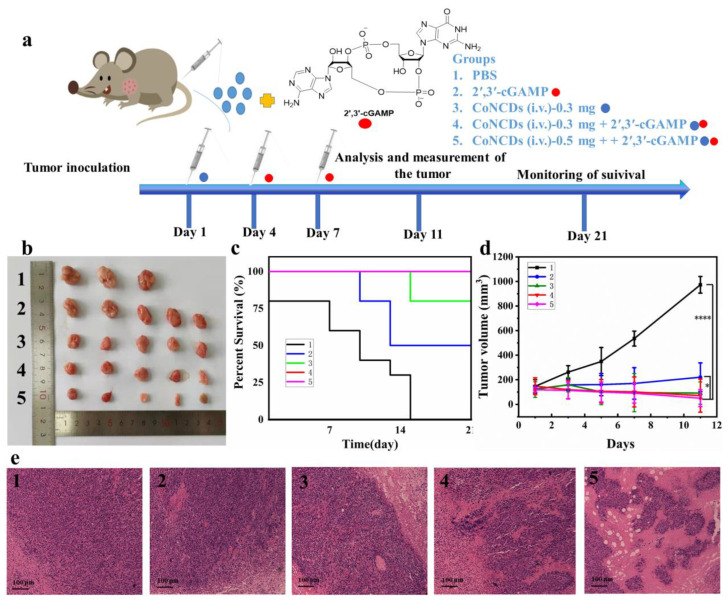
The therapeutic effect combined CoNCDs with 2′,3′-cGAMP for S180 tumor therapy: (**a**) schematic illustration of the experimental procedure; (**b**) picture of tumors in different treatments; (**c**) percent survival of S180 tumor-bearing mice after different treatments, the red line coincides with the pink line.; (**d**) tumor growth curves of different treatments; (**e**) tumor tissues of different groups on the 11th day were observed under a standard optical microscope (10 × 10) after H&E staining. **1**: PBS; **2**: 2′,3′-cGAMP (intravenously injected, 18 μg); **3**: CoNCDs were intravenously injected (CoNCDs = 0.3 mg); **4**: CoNCDs were intravenously injected (CoNCDs = 0.3 mg) plus 2′,3′-cGAMP (intravenously injected, 18 μg); and **5**: CoNCDs were intravenously injected (CoNCDs = 0.5 mg) plus 2′,3′-cGAMP (intravenously injected, 18 μg). * *p* < 0.05; **** *p* < 0.0001.

To assess the therapeutic effect of CoNCDs combined with 2′,3′-cGAMP therapy, an S180 tumor model was established (Figure 7a). The tumor-bearing mice were administered in five groups: (group **1**) PBS; (group **2**) 2′,3′-cGAMP (intravenously injected, 18 μg); (group **3**) CoNCDs were intravenously injected (CoNCDs = 0.3 mg); (group **4**) CoNCDs were intravenously injected (CoNCDs = 0.3 mg) plus 2′,3′-cGAMP (intravenously injected, 18 μg); and (group **5**) CoNCDs were intravenously injected (CoNCDs = 0.5 mg) plus 2′,3′-cGAMP (intravenously injected, 18 μg). Groups **3**–**5** were intravenously injected with CoNCDs on day 1. Subsequently, 2′,3′-cGAMP was intravenously injected into groups **2**, **4**, and **5** on day 4 and 7.

The tumor inhibition effects of CoNCDs were significantly stronger than that in the PBS and 2′,3′-cGAMP group. The photograph showed that the tumors in group 5 were small, in sharp contrast to those in groups **1** and **2** (Figure 7b). The use of CoNCDs alone showed the same antitumor activity in comparison to the in vivo antitumor effect of CoNCDs. The survival rate at day 21 (100%) of group **5** was much higher than that of the other groups (Figure 7c). Remarkably, from the tumor photographs (Figure 7b), tumor growth curves (Figure 7d), and tumor-bearing mice photographs (Figure S8), we found that tumors almost disappeared in group **5**; the volume of tumors was only 50.2 mm^3^. According to the results of group **2**, 2′,3′-cGAMP also had an anti-tumor effect on the S180 tumor model. 2′,3′-cGAMP activated the cGAS–STING pathway and induced the activation of the autoimmune system, resulting in a systemic anti-tumor effect. However, the therapeutic effect in group **2** was not as good as that in group **3** (CoNCDs used alone); the probable reason for this may be that CoNCDs had special properties of ROS production, had an effect on TME, and could kill tumor cells directly. Although 2′,3′-cGAMP used the immune system to kill tumor cells through a series of cascades, it exhibited a poor effect on solid tumors.

**Figure 8:**
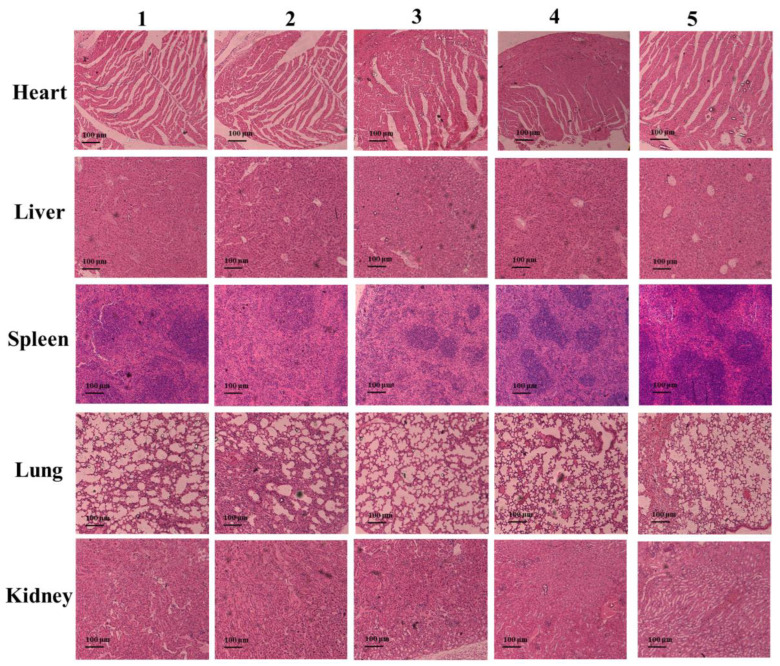
The H&E staining images of heart, liver, spleen, lung, and kidney tissues of mice after injection in different groups for 11 days (**1**. PBS; **2**. 2′,3′-cGAMP; **3**. CoNCDs (i.v.), 0.3 mg; **4**. CoNCDs (i.v.), 0.3 mg + 2′,3′-cGAMP; **5**. CoNCDs (i.v.), 0.5 mg + 2′,3′-cGAMP).

Among the five groups, there was no discernible variation in body weight (Figure S9). On the eleventh day following therapy, tumor tissue slices were H&E stained to examine the fate of the cancer cells and the harm to the major organs. According to the photos in Figure 7e, group **1** cancer cells maintained their normal cell structure and nuclear borders without any visible signs of necrosis or apoptosis. However, tumor cells displayed necrosis and ablation in groups **2**–**5**. Above all, the cancer cell apoptosis, cell membrane lysis, and tissue shriveling were most serious in group **5**. Additionally, no significant morphological alterations were seen in the major organs of groups **2**–**5** when compared to group **1** after H&E staining (Figure 8 and Figure S10).

To assess the immune response of the combined system, the levels of the cytokines tumor necrosis factor-α (TNF-α) and interferon-β (IFN-β) in the serum of mice cytokines were tested using an enzyme-linked immunosorbent assay (ELISA). The amount of TNF-α and IFN-β steadily rose after the injection of 2′,3′-cGAMP. TNF-α peaked at 24 h (Figure S11a), while IFN-β peaked at 8 h (Figure S11b). After 11 days, as we expected, the two cytokines produced after the injection of 2′,3′-cGAMP (groups **2**, **4**, and **5**) were higher than those produced by the non-injected groups (groups **1** and **3**) (Figure S11c,d). Additionally, group **5** had the greatest levels of TNF-α and IFN-β, demonstrating a systemic immune response against the tumor. At the same time, we found that the cytokines (TNF-α and IFN-β) were also increased in CoNCDs group (group **3**).

## 3. Discussion

From the characterization results of CoNCDs, we found that CoNCDs had good dispersion properties and uniform size, which render them advantageous for use in vivo [33,34,35]. Due to the presence of N-H on CoNCDs’ surface, CoNCDs exhibited a positive charge. Since the cell membrane was negatively charged, the positive CoNCDs could be easily adsorbed to the cell surface to play their role. CoNCDs exhibited a blue fluoresce and their excitation and emission wavelengths were 360 nm and 430 nm, respectively, indicating that CoNCDs had fluorescence properties and could achieve the imaging of cells; Figure 4 also confirms this result. We used OPD and H_2_O_2_ to detect the peroxidase-like activity of CoNCDs. The results showed that the CoNCDs had peroxidase properties and also confirmed that the type of ROS produced was •OH. As a powerful ROS, •OH could effectively kill cancer cells and act as a tumor treatment [36,37,38].

Nanozymes entered into tumor cells through endocytosis and then exerted peroxidase activity to produce ROS to kill cells; therefore, the cellular uptake efficiency was the basis of CoNCDs’ efficacy. CoNCDs can be taken up by cells because of the positive charge on the surface, which can be adsorbed to the cell surface and thus enter the cell. Intracellular ROS production was also confirmed.

After CoNCDs entered the cells, they began to kill the cell using its peroxidase-like activity. The results at the cellular level could also be observed as having a significant effect on cancer cells, which was because cancer cells had more hydrogen peroxide inside compared with normal cells [39,40], and we preliminarily explored its anti-tumor mechanism, which was realized by inducing apoptosis and inhibiting the cell cycle.

CoNCDs had good biocompatibility and could be further used for in vivo therapy. In this work, CoNCDs were used as a model nanozyme to evaluate the properties of the anti-tumor mechanism, and effective inhibition of S180 tumor was achieved. CoNCDs could significantly inhibit tumor growth, and no significant systemic toxicity was observed. In addition, TME limited immunotherapy effectiveness. Thus, based on CoNCDs’ good biocompatibility and therapeutic effect on tumors, we then introduced the cGAS–STING agonist. The combination of the CoNCDs and STING agonist significantly inhibited tumor growth, and no significant systemic toxicity was observed. Furthermore, the results of cytokines indicated that CoNCDs could kill cancer cells and cause the elevation of related cytokines through immune response. We speculated that this result was because the ROS generation induced the damage of DNA and stimulated the STING pathway.

Above all, the combined system achieved the enhanced tumor synergistic immunotherapy through TME reprogramming via the peroxidase-like activity of the CoNCDs and cGAS–STING signaling pathway agonist synergistically. Our work provides not only a new effective way to reprogram TME in vivo, but also a promising synergic antitumor therapy strategy.

## 4. Materials and Methods

Co(NO_3_)_2_·6H_2_O, EDTA-Na, and methyl alcohol were obtained from Sigma (St. Louis, MO, USA). 1,2-diaminobenzene (OPD) and hydrogen peroxide (H_2_O_2_, 30%) were obtained from J&K China Chemical Ltd. (Shanghai, China). A 2′,7′-dichlorofluorescin diacetate (DCFH-DA) Kit and Live/Dead Cell Staining Kit (Calcein-AM/PI) were obtained from Sigma. Cells were purchased from the national collection of authenticated cell cultures. A CycleTEST™ Plus DNA Reagent Kit and PE Annexin V Apoptosis Detection Kit were obtained from BD Biosciences (Franklin Lakes, NJ, USA). The BALB/c mice (4~5 weeks old, 18~20 g) were obtained from Hualan Biology. The animal experiments were approved by the Use Committees of Henan Normal University.

### 4.1. Synthesis of CoNCDs

First, 8.8 g of EDTA-Na and 14.0 g of Co(NO_3_)_2_·6H_2_O were added to 40 mL of water and stirred until dissolved completely. Then, 20 mL of methyl alcohol was added and stirred for 1 h at 25 °C. The obtained pink precipitate was dried at 60 °C, and it was calcined in a tube furnace at 300 °C (heating rate of 5 °C/min) for 2.5 h under N_2_ exposure. Then, 0.5 g of brown sample was dissolved in 20 mL of water, sonicated for 2 h, and centrifuged at 15,000 rpm for 15 min. The final product was obtained after dialysis in a dialysis bag (Da MW cut-off 1000) for 2 days. We used ICP and HPLC to monitor the impurities in extracalytic solution, and the results are shown in Figure S12. CoNCDs could be enough for proper purification after 2 days of dialysis. Subsequently, the obtained solution was lyophilized. We performed 5 replicate syntheses for the CoNCDs.

### 4.2. Characterization

The morphology and size of CoNCDs were used in JEM-2100 transmission electron microscopy (TEM). X-ray photoelectron spectroscopy (XPS, Thermo-ESCALAB250Xi spectrometer with Al Kα radiation of 1486.6 eV and 1253.6 eV) and a Nicolet iS10 FTIR spectrophotometer were used to analyze the chemical state of CoNCDs. Confocal laser scanning microscopy (CLSM, Olympus, FV1200-MPE) (Tokyo, Japan) was employed to observe cell uptake of CoNCDs. Flow cytometry (FCM, FACSCalibur^TM^, BD Biosciences) was used to observe cells’ cycle and apoptosis.

### 4.3. Cytotoxic Effect of CoNCDs

An MTT assay of MCF-7, Hela, A375, S180, 293T, 3a cell line was carried out to detect the cytotoxic effect of CoNCDs (0.01–1 mg/mL) at 24 h.

Live/dead cell staining needed 10,000 HeLa cells per dish. After 24 h of incubation, the prepared suspension of CoNCDs (250 μg/mL and 500 μg/mL) was added to the corresponding wells. After treatment with CoNCDs as described above for 10 h, the cells were stained with Calcein (AM, 2 μg/mL, 200 μL per dish) and Propidium Iodide (PI, 10 μg/mL, 200 μL per dish) at 37 °C for 15 min and washed three times. Cellular fluorescence images were captured using CLSM.

### 4.4. Cells’ Apoptosis and Cycle Effect of CoNCDs

To evaluate the apoptosis of well-prepared CoNCDs, 10,000 Hela cells were incubated with CoNCDs (200 μg/mL) per dish for 6 h. The following protocol was based on the instruction in the corresponding kit, and then analyzed using FCM.

To evaluate the cell cycle effect of CoNCDs, 10,000 Hela cells were incubated with CoNCDs (250 μg/mL) per dish for 6 h. We followed the suggested protocol using the corresponding kit and then analyzed using FCM.

### 4.5. Detection of Cellular Uptake and Intracellular ROS Level of CoNCDs

A total of 10,000 HeLa cells were cultivated in the glass-bottom culture dishes to assess the imaging of cellular uptake in vitro. The HeLa cells were exposed to CoNCDs (200 μg/mL) for 0.25, 0.5, 1, 2, 4, and 8 h to observe cellular absorption. The excitation and emission wavelengths were 430 and 560 nm, respectively.

HeLa cells were examined using an ROS kit. CoNCDs (100 μg/mL, 1 mL in each well) were treated with HeLa cells (8 × 10^3^ cells/dish) in confocal cell culture dishes for 6 h. Thereafter, the cells were washed with pre-cooling PBS, and DCFH-DA was added (0.01 mM, 1 mL per well) for 30 min in the dark, in an attempt to detect intracellular ROS levels. A fluorescence microscope was used to observe the wavelengths of 488 nm and 525 nm for excitation and emission, respectively. Rosup was used as a control.

### 4.6. Hemolytic Properties of CoNCDs

Erythrocytes were collected from mouse blood and diluted to a final concentration of 1% (*v*/*v*) with PBS. Aliquots of the erythrocyte suspension (100 μL) were added into the wells of a sterile 96-well plate. Different concentrations of 2.0 μL of CoNCDs suspension were used with different concentrations in PBS. Then, samples were centrifuged for 10 min at 3500 rpm after being incubated at 37 °C for 30 min with gentle shaking. A different sterile 96-well plate with 50 μL of PBS buffer in each well was used to receive aliquots (50 μL) of the supernatant. At 543 nm, hemoglobin release was observed using a microtiter plate reader. Complete hemolysis was achieved by the erythrocytes incubated with 1% Triton X-100. Percentage hemolysis was computed using the following formula:

percentage hemolysis = (OD_CoNCDs_ − OD_PBS_)/(OD_complete hemolysis_ − OD_PBS_) × 100%

### 4.7. Biocompatibility of CoNCDs

Animal experiments were approved by the Use Committees of Henan Normal University (serial number: HNSD-2023BS0303). Normal mice were selected as experimental subjects, and the mice were divided into two groups: group **1**: PBS; group **2**: CoNCDs (0.5 mg). The mice were sacrificed at 4, 12, 24 h, and 2 days following intravenous injection of CoNCDs (1 mg/mL). The heart, liver, spleen, lung, and kidney were removed; then, all the organs were digested with HNO_3_, and the contents of Co in each organ were detected via ICP-MS. Then, the organs of mice sacrificed after 2 days were sliced for HE staining.

### 4.8. Anti-Tumor Effect of CoNCDs In Vivo

2 × 10^6^ S180 cells dispersed in 50 μL of PBS buffer were injected subcutaneously into female BALB/c mice. When the volume increased to 100~200 mm^3^, mice were categorized into 4 groups (3 mice per group): PBS (100 μL); CoNCDs (intratumoral injection, injection dose = 50 μL, 300 μg); CoNCDs (intravenous injection, injection dose = 50 μL, 300 μg); and CoNCDs (intravenous injection, injection dose = 50 μL, 500 μg). CoNCDs were injected on the first day after tumor implantation.

Every two days, the tumor volume and body weight were assessed. The tumor volume was determined using the formula V = a × b^2^/2, where a stands for its length and b for its width. According to ethical requirements, the mice were euthanized when tumors reached 1000 mm^3^. The collected tumor tissues and the tumor sizes in various groups were then compared.

### 4.9. Antitumor Immunotherapeutic of CoNCDs Combined 2′,3′-cGAMP In Vivo

Mice were assigned to 5 groups (10 mice per group) as follows: PBS (intravenous injection, 100 μL); 2′,3′-cGAMP (intravenous injection, injection dose = 100 μL, 18 μg); CoNCDs (intravenous injection, injection dose = 100 μL, 300 μg); CoNCDs (intravenous injection, injection dose = 50 μL, 300 μg) + 2′,3′-cGAMP (intravenous injection, injection dose = 50 μL, 18 μg); and CoNCDs (intravenous injection, injection dose = 50 μL, 500 μg) + 2′,3′-cGAMP (intravenous injection, injection dose = 100 μL, 18 μg). To assess the in vivo anti-tumor effects mediated by the CoNCDs, 3 mice were randomly selected and sacrificed and dissected after the tumors reached 1000 mm^3^. The remaining mice were kept for 3 weeks, and their survival rate was recorded.

## 5. Conclusions

In summary, we have successfully developed a synergy antitumor therapy strategy: nanozymes combined with the cGAS–STING signaling pathway agonist producing oxidative damage-mediated systemic innate immunity to remodel TEM for solid tumor S180 anti-cancer therapy. The combined system achieved enhanced tumor synergistic immunotherapy through TME reprogramming via the peroxidase-like activity of the CoNCDs and cGAS–STING signaling pathway agonist synergistically. Overall, our work provides not only a new effective way to reprogram TME in vivo, but also a novel strategy to achieve whole-body therapeutic responses with nanozymes and the cGAS–STING signaling pathway for cancer immunotherapy.

## Data Availability

Data will be provided on request.

## Supplementary Materials

The following supporting information can be downloaded at: https://www.mdpi.com/article/10.3390/ijms241813935/s1. References [41,42] are cited in the supplementary materials.
