# Supplementary material for: Remodeling of Tumor Microenvironment by Nanozyme Combined cGAS–STING Signaling Pathway Agonist for Enhancing Cancer Immunotherapy
Source: Int J Mol Sci. 2023 Sep 11;24(18):13935. doi: 10.3390/ijms241813935 (PMC10530945; doi:10.3390/ijms241813935)
Supplement: Supplementary file 1 [file ijms-24-13935-s001.zip › ijms-2574954-supplementary.pdf]

Supplementary Information

Remodeling of Tumor Microenvironment by Nanozyme Combined cGAS–STING Signaling Pathway Agonist for Enhancing Cancer Immunotherapy

Wenpei Dong [1,2], Mengting Chen [1,2], Chun Chang [1,2], Tao Jiang [1,2], Li Su [1,2], Changpo Chen [1,2,*] and Guisheng Zhang [1,2,*]

[1] Electronic S Collaborative Innovation Center of Henan Province for Green Manufacturing of Fine Chemicals, Key Laboratory of Green Chemical Media and Reactions, Ministry of Education, Henan Normal University, Xinxiang 453007, China; dongwenpei@htu.edu.cn (W.D.); jiangtao@htu.edu.cn (T.J.); suli@htu.edu.cn (L.S.)

[2] Henan Key Laboratory of Green Chemical Media and Reactions, School of Chemistry and Chemical Engineering, Henan Normal University, Xinxiang 453007, China

* Correspondence: chenchangpo@htu.edu.cn (C.C.); zgs@htu.cn (G.Z.)

Table of contents

1. Experimental Sections……………………………………………………… (S3)
1.1 Reagents and Materials.

1.2 Kinetic Analysis

1.3 Histological analysis

1.4 Cytokine detection

1.5 Statistical methods

2. Supplementary Figures and Tables………………………………………. (S4-S16)

Figure S1. The photo of CoNCDs dispersed in water.

Figure S2. The Zeta potential of CoNCDs (1mg/mL).

Figure S3. The XRD pattern of CoNCDs.

Figure S4. The UV–Vis absorption spectra of CoNCDs.

Figure S5. The fluorescence emission spectra of CoNCDs.

Figure S6. Biocompatibility of CoNCDs.

Figure S7. Picture of mice in different treatments.

Figure S8. Average body weights of mice in different treatments.

Figure S9. Tumor weight of different treatments.

Figure S10. The pictures of heart, liver, spleen, lung, and kidney tissues of mice after subcutaneous injection in different groups for 11 days.

Figure S11. Cytokine profiles in mice treated with CoNCDs and 2′,3′-cGAMP.

Figure S12. The HPLC results of the external dialysis solution. Left: the liquid chromatogram of HPLC; Right: the peak area of 5 peaks.

Table S1. Comparison of the kinetic parameters of CoNCDs in different pH7.

Table S2. The IC_{50} values.

3. Scientific Research Experiment Ethics Review Approval…………………(S17-S18)

1. Experimental Sections

1.1 Reagents and Materials

Hydrogen peroxide (H_2O_2, 30%), $NaNO_3$, $NaNO_2$, and $FeCl_3 \cdot 6H_2O$ were purchased from Tianjin Deen Chemical Reagent Co., Ltd. (Tianjin, China).

1.2 Kinetic Analysis

Kinetic measurements were conducted at 37 °C in a 400 μL micro cuvette. In total, 100 μL of CoNCDs solution (12.5 μg mL^{-1}), 100 μL of OPD (10 mM), and 100 μL of H_2O_2 (10 mM) was incubated in a cuvette for 10 min using a TU-1900 double-beam UV-vis spectrophotometer at 420 nm monitoring the absorbance change in the time course mode. The kinetics data were obtained by varying the concentration of one substrate of H_2O_2 or OPD while keeping the other's concentration constant. The Michaelis–Menten constant was calculated using the Lineweaver–Burk plot.

1.3 Histological analysis

H&E staining: On the 21st day after treatment, major organs (heart, liver, spleen, lung, and kidney) and tumors were dissected, rinsed with PBS, fixed in 4% formaldehyde, embedded in paraffin, and sectioned into 5 μm slices, and a pathological examination was performed after hematoxylin and eosin staining.

1.4 Cytokine detection

The plasma concentrations of IFN-β and TNF-α were measured using an ELISA kit.

1.5 Statistical methods

The data of the experiments were presented as mean ± Standard Deviation (SD). The significant difference was analyzed via one-way ANOVA followed by Tukey's post hoc test.

2. Supplementary Figures

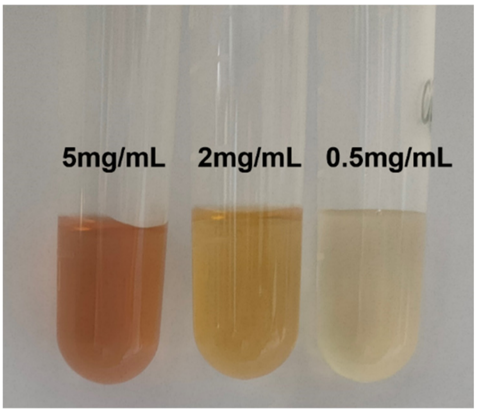

Figure S1. Photo of CoNCDs dispersed in water.

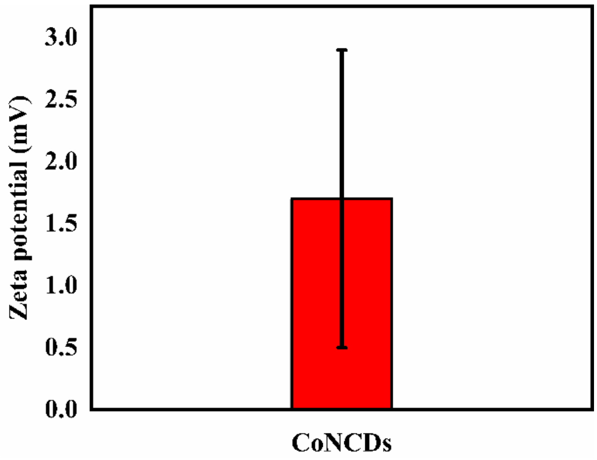

Figure S2. The Zeta potential of CoNCDs (1mg/mL).

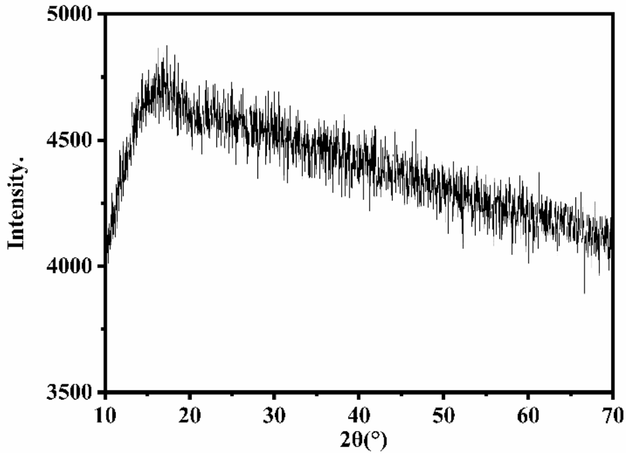

Figure S3. The XRD pattern of CoNCDs.

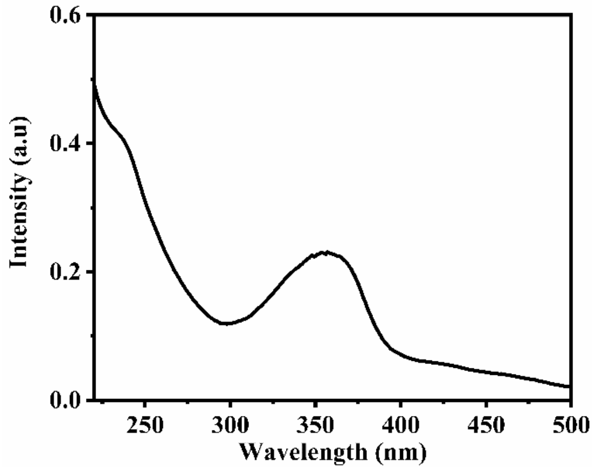

Figure S4. The UV–Vis absorption spectra of CoNCDs.

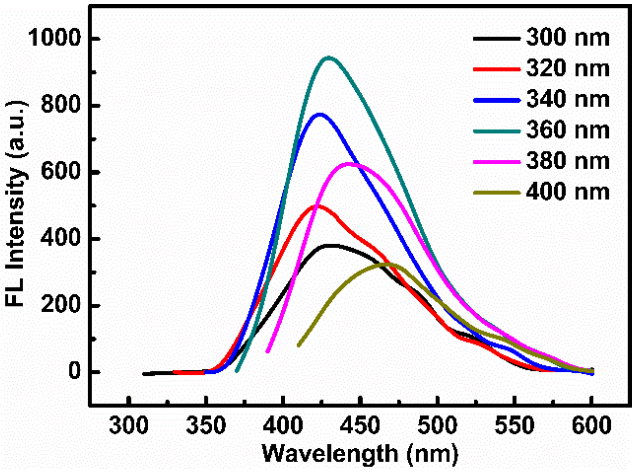

Figure S5. The fluorescence emission spectra of CoNCDs.

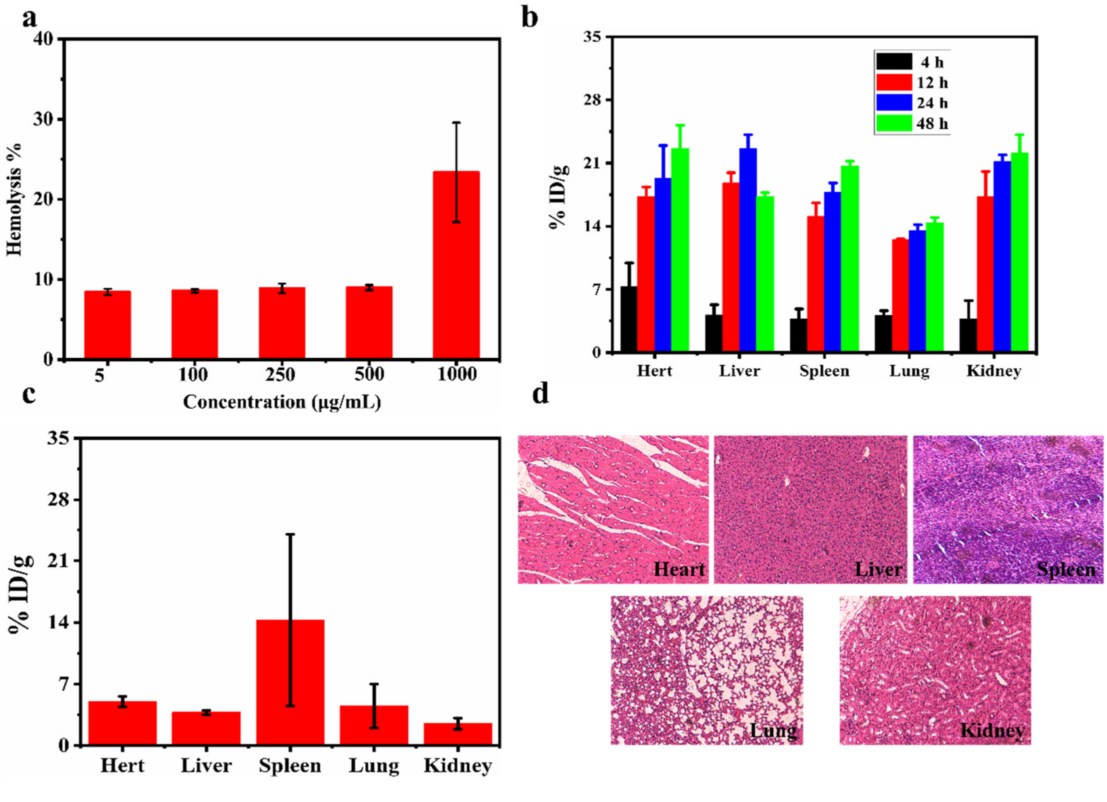

Figure S6. Biocompatibility of CoNCDs: (a) the hemolytic rates of CoNCDs; (b-c) the biodistribution of PBS via ICP-MS analysis; (d) all these organs are observed under a standard optical microscope after hematoxylin and eosin (H&E) staining.

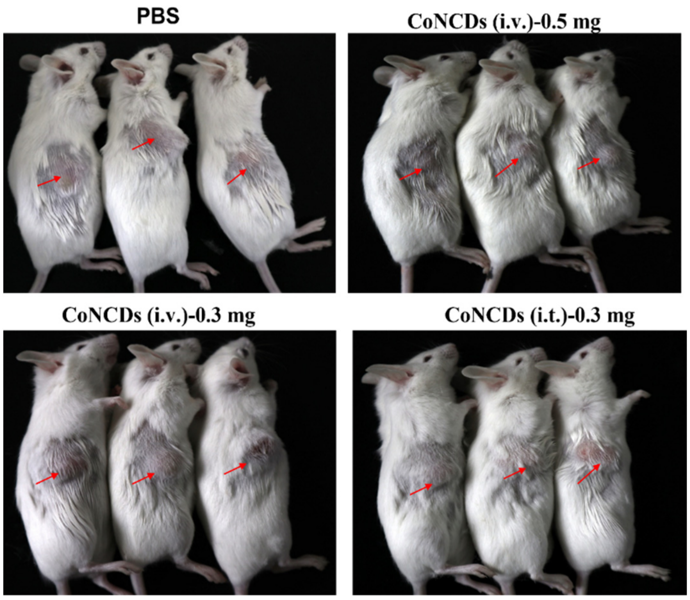

Figure S7. Picture of mice in different treatments.

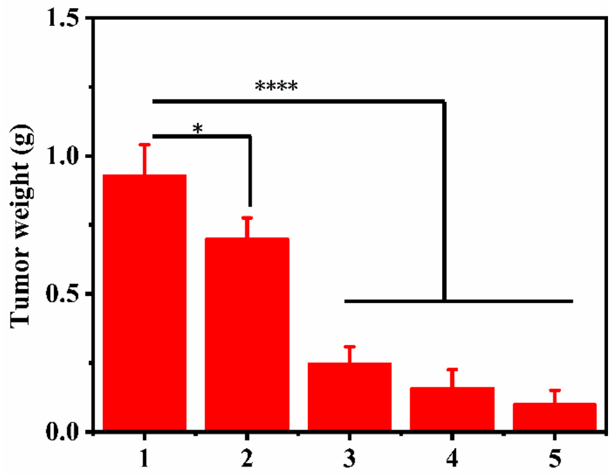

Figure S8. Tumor weight of different treatments, * $p < 0.05$, **** $p < 0.0001$.

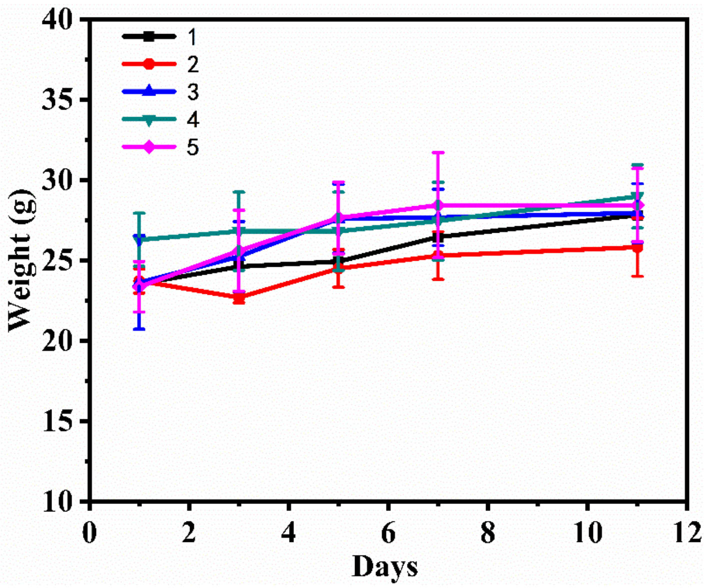

Figure S9. Average body weights of mice in different treatments.

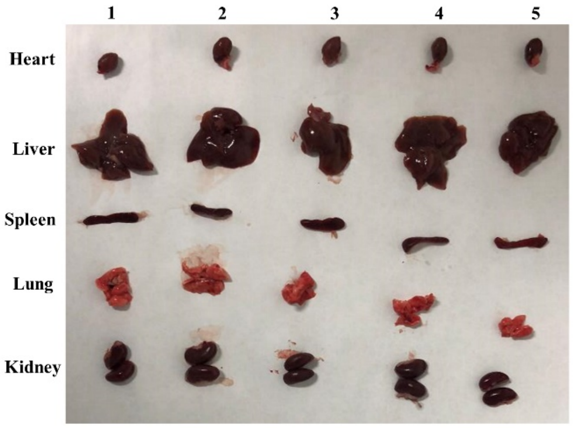

Figure S10. The pictures of heart, liver, spleen, lung, and kidney tissues of mice after subcutaneous injection in different groups for 11 days (1. PBS; 2. 2′,3′-cGAMP; 3. CoNCDs -0.3 mg; 4. CoNCDs -0.3 mg + 2′,3′-cGAMP; 5. CoNCDs -0.5 mg + 2′,3′-cGAMP).

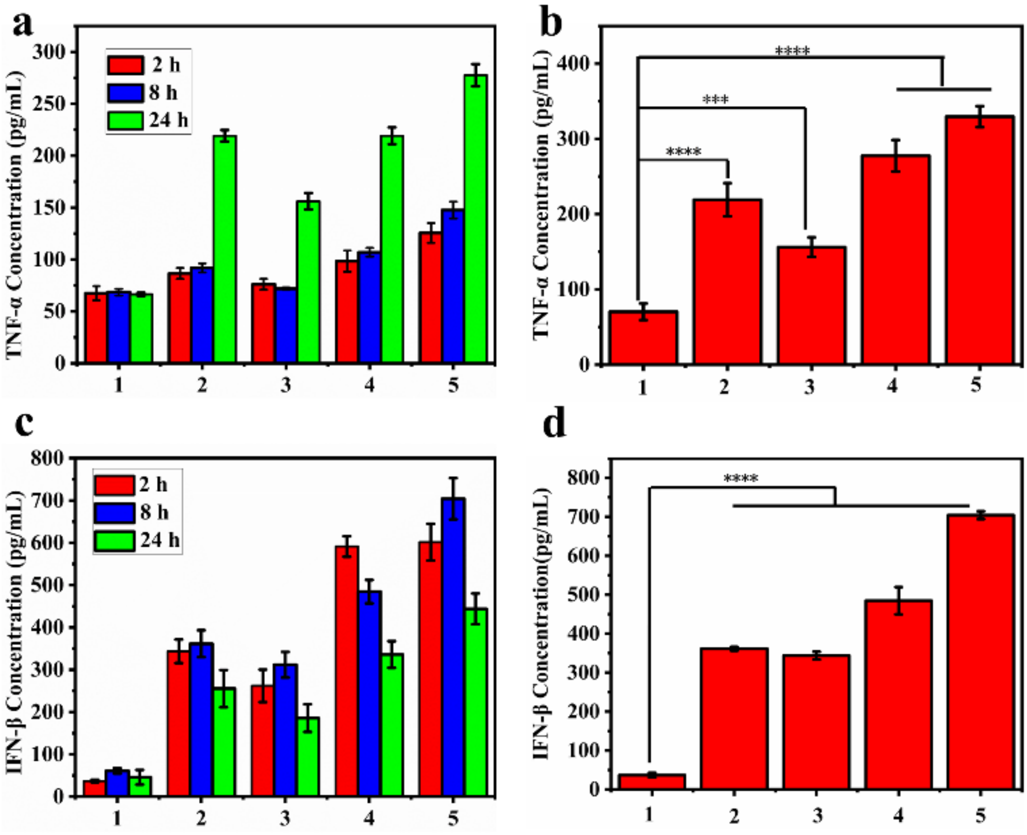

Figure S11. Cytokine profiles in mice treated with CoNCDs and 2′,3′-cGAMP: (a) amounts of TNF-*α* were estimated via ELISA at 2, 8, and 24 h; (b) amounts of TNF-*α* were estimated via ELISA on the 11th day; (c) amounts of IFN-β were estimated via ELISA at 2, 8, and 24 h; (d) amounts of IFN-β were estimated via ELISA on the 11th day (b). ***P < 0.001; ****P < 0.0001 (1. PBS; 2. 2′,3′-cGAMP; 3. CoNCDs (i.v.)-0.3 mg; 4. CoNCDs (i.v.)-0.3 mg + 2′,3′-cGAMP; 5. CoNCDs (i.v.)-0.5 mg + 2′,3′-cGAMP).

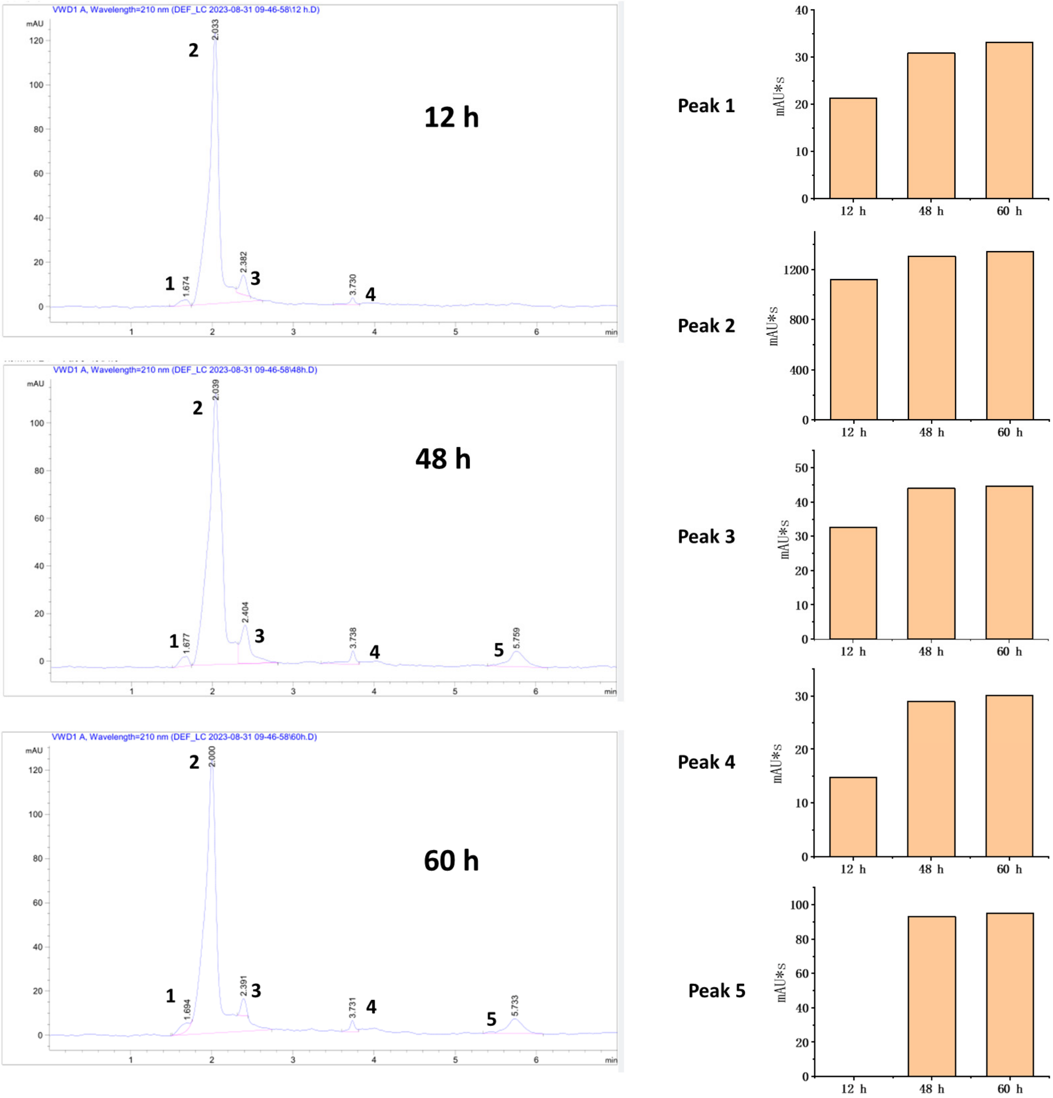

Figure S12. The HPLC results of the external dialysis solution. Left: the liquid chromatogram of HPLC; right: the peak area of five peaks.

.

Supplementary Tables

Table S1. Comparison of the kinetic parameters of CoNCDs in different pH.

pH values	Substance	K_m/mM	$V_{max}/10^{-8}$ M·s^{-1}
pH=7	OPD	1.06	21.5
	H_2O_2	1.26	17.8

Table S2. The IC_{50} values.

	Cells	IC_{50} (mg/mL)
CoNCDs	3a	1.528±0.042
	293T	1.156±0.051
	A375	0.282±0.027
	Hela	0.209±0.013
	MCF-7	0.187±0.030
	S180	0.185±0.062
Co-CDs	A549[1]	0.256
MOF-Pt(IV)	4T1[2]	0.125

References

1. Lu, W.; Guo, Y.; Zhang, J.; Yue, Y.; Fan, L.; Li, F.; Dong, C.; Shuang, S. A High Catalytic Activity Nanozyme Based on Cobalt-Doped Carbon Dots for Biosensor and Anticancer Cell Effect. *ACS Appl. Mater. Interfaces* **2022**, *14*, 57206–57214.
2. Wu, P.H.; Cheng, P.F.; Kaveevivitchai, W.; Chen, T.H. MOF-based nanozyme grafted with cooperative Pt(IV) prodrug for synergistic anticancer therapy. *Colloids Surf. B Biointerfaces* **2023**, *225*, 113264.

3. Scientific Research Experiment Ethics Review Approval

Henan Normal University Scientific Research Experiment Ethics Review Approval Form

Serial number：HNSD—2023 BS 030[illegible]

Project Title	Synthesis, bioactivity and target identification of cyclic oligonucleotides in mouse and human immune cells		
Fund Source	National Natural Science Foundation of China（22277022）		
Principle Investigator	Changpo Chen	Department	School of Chemistry and Chemical Engineering
Review category	☑Apply for animal experiment<br>□Scientific research project declaration □others		

(Summary of the research content of the project and associated ethical objects and experimental plans)

This project uses BALB/c mice mice as experimental materials, which requires establishments of S180 and CT26 tumor models and collection of animal tissues. The research work in this area involves the ethics of animal experiments. In order to alleviate the pain of experimental animals, anesthetics are applied before the biopsy; to prevent infection and death during surgical dissection and blood draw, various surgical instruments and micro syringes will be disinfected; mice are raised in clean animal rooms and fed with feed and drinking water in time; intravenous blood draws use disposable syringes, the needle angle is appropriate to prevent injury to internal organs after the needle is inserted; the mice are euthanized after the experiment, confirm After the death of the animal, the carcass is burned in a concentrated manner to prevent environmental pollution.

The applicant (project principal) promises：

The above information is true. If approved, I will conduct research in strict accordance with the provided plan, and abide by the scientific research experiment ethics code and related regulations, and voluntarily accept the supervision and inspection of the school academic committee. If the regulations are violated, I will voluntarily accept corresponding punishments.

Project principal signature [signature] Date 2023. 1.8

Review opinions of the Academic Committee of the College:

After review by the Academic Committee of School of Chemistry and Chemical Engineering, the design specifications, research content and process of the project meet the ethical requirements of the relevant scientific research experiments promulgated by the state, and it is agreed that the project will be implemented as planned.

Academic Committee o School of Chemistry and Chemical Engineering (seal)

Date 2023.1.8

Review opinions of the school academic committee:

1、Applicant qualifications：	☑Compliance	☐Non-compliant	
2、Experimental program：	☑Appropriate	☐Inappropriate	
3、Review conclusion：	☑Agree	☐Discuss after revision	☐Disagree

Academic Committee of Henan Normal University (seal)

Date 2023.1.8

Notes for filling in the form:

1. The project name and research information are consistent with the final version of the declared project to avoid unnecessary trouble;
2. The ethical objects are the experimental objects specifically related to ethics in the project research institute. The experimental plan includes the reasons why ethical objects must be used, the experimental process and protective measures, and the processing methods after the end of the experiment;
3. The seal of the academic committee of the college can be replaced by the college;
4. When applying, please submit this form in duplicate and the electronic version, and the serial number shall be filled in by the academic committee.
